# Timeliness: The Authors' Vested Right but the Editors' Last Concern 

**Published:** 2016-12-08

**Authors:** Mostafa Rad, Abdolghader Assarroudi, Mohammad Reza Armat, Nematullah Shomoossi

**Affiliations:** ^a^ School of Nursing, Sabzevar University of Medical Sciences, Sabzevar, Iran; ^b^ School of Nursing, Mashhad University of Medical Sciences, Iran; ^c^ School of Medicine, Sabzevar University of Medical Sciences, Sabzevar, Iran

## Dear Editor-in-Chief

Practiced in various journals, peer reviewing is undoubtedly the core of science (as opposed to non-science) production in all disciplines, particularly in medical sciences due to their direct and immediate effect on human health. Publication provides the chance for bits of knowledge to get integrated into the body of global knowledge and to share it with other researchers^1^. This involves the reviewing of a submitted manuscripts, which varies from journal to journal; however, the most common steps appear to be the notice of receipt to the author, primary appraisal by the editor-in-chief or the editorial team, extending the early impressions to the corresponding author, re-submission of the manuscript by the author, sending out the manuscript to external reviewers or its immediate rejection, receiving the reviewer’s comments, finalizing the comments, rejection or sending the comments to the author for revising the manuscript, re-submission of the revised version, and the final decision to accept or reject it. What comes to be important at this stage is how the journal interacts with the corresponding author.

From an interactive perspective, mutual rights are considered for both the journal and the authors; also, other stakeholders including the reviewers, readers, and the journal staff all hold rights in the publication process ,^3^. For instance, journals demand authors not to submit the manuscript to other journals while it is being considered for publication, not to have it published elsewhere, and to precisely format the manuscript by the rules of the intended journal ^4^. In return, authors expect a fair reviewing process and timeliness – which is not considered oftentimes. Stressing this concern, ICMJE puts it as follows: "*Editors should do all they can to ensure timely processing of manuscripts with the resources available to them. If editors intend to publish a manuscript, they should attempt to do so in a timely manner and any planned delays should be negotiated with the authors. If a journal has no intention of proceeding with a manuscript, editors should endeavor to reject the manuscript as soon as possible to allow authors to submit to a different journal*" ^3^.

All in all, we found this concern as a major violation of the authors’ rights by journals where editors place timeliness as the last priority^4^ for particular reasons. Moreover, authors complain of wastage of time, dissatisfaction, discouragement, losing the chance to prompt publication of their innovative findings, and losses due to (non) financial grounds^5-7^. Ignoring timeliness by journal editors may originate in either of the above-mentioned steps, which contribute to journals’ falling behind schedules for reasons such as lack of rapid reviewers, slow process of reviewing, lack of cooperation of authors in responding to revisions ^8^ , language problems despite acceptable scientific results reported^9^, and so on. Therefore, editors-in-chief must be requested, or expected, to re-consider their undeniable role – either directly or indirectly – in supervising all publication stages ^10^. This expectation may not be considered as unlikely because authors have already made their share in science production by doing the research and reporting it; what remains is the delicately speeding up the publication process, which is the share of editors.

While the authors’ rights of timeliness (in order to help speed up the publication process as well as attaining higher rates of satisfaction), provisional and permanent measures may be put forward below to help bridging the gap between the authors’ expectations and editors’ amenities:

a) Employing energetic, brisk and agile experts for journalism in order to accelerate the message transmission and extending the editors’ views and decisions to authors,
b) Portraying an appropriate and logical timetable for each publication stage and insisting on the commitment to care for timeliness,
c) Activating the submission system in order to supervise reviewing and deadlines,
d) Exercising rewarding policies for committed reviewers who provide feedback before deadlines,
e) Rapid reviewing options, and
f) Rapid but respectful rejection if the manuscript does not fall within the scope of the journal, or if it is not eligible for peer reviewing, and providing acceptable reasons for the author at the earliest possible time ^11^.

Mostafa Rad (PhD)^a^, Abdolghader Assarroudi (MSc)^a^, Mohammad Reza Armat (MSc)^b^, Nematullah Shomoossi (PhD)^c^*

^a^ *School of Nursing, Sabzevar University of Medical Sciences, Sabzevar, Iran*

^b^ *School of Nursing, Mashhad University of Medical Sciences, Iran*

^c^ *School of Medicine, Sabzevar University of Medical Sciences, Sabzevar, Iran*

*****Correspondence to:***** *Nematullah Shomoossi (PhD)*

***E-mail:*** nshomoossi@yahoo.com
